# Implementing patient–public engagement for improved health: Lessons from three Ghanaian community‐based programmes

**DOI:** 10.1111/hex.13866

**Published:** 2023-09-11

**Authors:** Samuel E. Ankomah, Adam Fusheini, Sarah Derrett

**Affiliations:** ^1^ Department of Preventive and Social Medicine University of Otago Dunedin New Zealand; ^2^ Ngāi Tahu Māori Health Research Unit, Division of Health Sciences University of Otago Dunedin New Zealand

**Keywords:** community‐based health interventions, community engagement, Ghana, patient–public engagement, Sub‐Saharan Africa

## Abstract

**Background:**

Community‐based health interventions have been implemented as a key strategy for achieving improved health outcomes in Ghana. Effectiveness, however, largely depends on the successful implementation of patient–public engagement (PPE). Although several PPE studies have been conducted in Ghana, little research has been done to understand the specific role of PPE in the context of implementing community‐based health programmes. This paper, therefore, examines the extent of PPE implementation in three selected community‐based health programmes (Community‐based Health Planning and Service [CHPS], Community‐based Maternal and Child Health and Buruli Ulcer) to understand their specific effects on health outcomes.

**Methods:**

Three focus groups, involving 26 participants, were held in three districts of the Ashanti region of Ghana. Participants were mainly health service users involving community health committee members/volunteers, residents and health professionals. They were invited to participate based on their roles in the design and implementation of the programmes. Participants focused on each of Rifkin's spider‐gram components. Data were transcribed and analysed descriptively using NVIVO 12 Plus.

**Results:**

PPE implementation was found to be extensive across the three programmes in specific areas such as organisation and resource mobilisation. PPE was more restricted in relation to community needs assessment, leadership and management, particularly for the CHPS and Buruli Ulcer programmes.

**Conclusion:**

Findings suggest that benefits from community‐based health interventions are likely to be greater if PPE can be widely implemented across all dimensions of the spider‐gram framework.

## 1 BACKGROUND

Globally, there has been strong advocacy for shifting the focus of policy development and system improvement from an elitist model of expert advice being the authoritative source for policy design to ensuring lay experts or citizens have a ‘voice’ in shaping policies and decisions.^1^, ^2^ As advocated by the 1978 Alma‐Ata declaration on primary healthcare, people have the right and duty to participate in the planning and implementation of healthcare strategies.^3^, ^4^ Participation can provide wider community acceptance of health interventions and services responsive to the needs of the people—thereby supporting improved outcomes.^5^

In many Sub‐Saharan African countries, including Ghana, community‐based health interventions have been implemented to achieve improved health outcomes. The interventions have focused on improving outcomes for neglected tropical diseases,^6^ malarial infections^7^, ^8^, ^9^ and maternal health outcomes.^10^, ^11^ While these interventions have achieved some successes, there are still concerns about poor outcomes.^12^, ^13^ Recent studies evaluating outcomes of community‐based health interventions have called for greater patient–public engagement (PPE) in the design and implementation of such health programmes to improve outcomes.^13^, ^14^, ^15^ The World Health Organisation also highlights PPE as one of the nine key priority areas for improving health outcomes in Africa.^16^, ^17^ Thus, PPE has been prioritised in the national health policies of many African countries.^17^ For instance, in Ghana, two major healthcare policies: the National Health Policy and the National Healthcare Quality Strategy have prioritised PPE as key to improving health outcomes. The Ghanaian health system also provides legislation to support the implementation of PPE across all levels of the health system.^18^

Despite the demonstrated advantages, anecdotal evidence in Ghana still suggests low (or no) PPE as a possible reason for unsuccessful outcomes in community‐based health interventions.^15^, ^19^ Research in Ghana does not appear to have investigated the specific role of PPE in the context of implementing community‐based health programmes. A study conducted in the Upper West region of Ghana^20^ assessed a community's involvement in a national Community‐based Health Planning and Services (CHPS) programme but not the extent of PPE during the implementation of programmes. This research, therefore, aims to examine PPE in the implementation of three selected community‐based health interventions, and PPE's effects on the programme goals.

## 2 METHODS

### 2.1 Study setting

This research was conducted in Ghana, a West African country sharing borders with Burkina Faso to the north, Côte d'Ivoire to the west and Togo to the east; the southern border is formed by the Gulf of Guinea coastline.^21^ Specifically, the study was located in three districts in the Ashanti region of Ghana: Afigya‐Kwabre South, Sekyere South and the Asante‐Akim North Districts. These districts are largely rural and have a combined population of approximately 440,000 people living over a wide geographical area (approximately 1991 km^2^).^22^, ^23^, ^24^ Most residents are peasant farmers with low incomes, farming food crops such as maize, cassava, plantains, yams, citrus and vegetables for family consumption.^23^ The majority of residents belong to the Ashanti ethnic group and Twi is the main language spoken.

In terms of healthcare delivery, the Ministry of Health is administratively responsible for the provision of overall health policy in Ghana, particularly in areas of institutional development, coordinating with key partners, stakeholders and other agencies in ensuring high‐quality performance and accountability.^25^ Administratively and functionally, Ghana has a five‐tier health system operating at national, regional, district, subdistrict and community levels.^26^ The national level develops policies intended to be implemented by the regional health directorates in the various districts, subdistricts and community levels. The regional levels supervise healthcare delivery at the district level as well as mobilise and allocate resources for effective healthcare delivery at the districts, subdistricts and community levels. The districts and subdistricts also implement healthcare policies and provide healthcare services at the local levels.

### 2.2 Design and sampling

The research described in this paper was part of a broader qualitative case study project that focused on public–patient engagement for improving the performance of health systems using the case of Ghana. The selection of study sites included a survey conducted among health service administrators in the Ashanti region of Ghana to identify districts and communities with good PPE structures. The wider case study included individual interviews and document analysis focused on understanding the overall functioning of PPE in three districts. This paper is focused on findings from a focus group undertaken in each of the three districts. A different community‐based health intervention programme was selected from each district to understand the extent to which the community was involved in the design and implementation and how this influenced the programme goals. The three community‐based health interventions were: CHPS, Community‐based Maternal and Child Health (CMCH) and a Buruli Ulcer programme.

CHPS is a national primary healthcare strategy aimed at mobilising grassroot community resources and leadership to reduce health inequalities and remove geographical and physical barriers to healthcare access in Ghana—particularly for lower‐income populations^27^ and underserved communities. One district had implemented this programme to specifically improve a community's access to healthcare services. The CMCH programme was implemented as a local initiative in the second selected district to address worsening maternal and child health outcomes in a rural community. Lastly, the Buruli Ulcer programme was implemented in the third district as an urgent approach was required to control the increased spread of the disease.

Two groups of participants were involved in each focus group: health service providers (including nurses, midwives, public health officers, programme representatives etc.); and users (including Community Health Management Committee (CHMC) members, Community Health Volunteers (CHVs), assembly members, traditional leaders and other opinion leaders). The research employed maximum variation sampling to purposively invite participants based on their roles within the health programme being implemented. Table 1 shows the range of participants.

**Table 1:** Distribution of study participants.

	CHPS programme	CMCH programme	Buruli ulcer
Queen mother/traditional leader	1	1	1
Resident/opinion leader	1	1	1
Community health nurse/public health officer/midwife	2	1	1
CHMC & CHV	4	3	2
Assemblyman/assemblywoman	0	1	0
Community health volunteer only	1	1	1
Programme/community health planning coordinator	1	1	1
Total	10	9	7

Abbreviation: CHMC, Community Health Management Committee; CMCH, Community‐based Maternal and Child Health; CHPS, Community‐based Health Planning and Services; CHV, Community Health Volunteer.

### 2.3 Data collection and participants' characteristics

The three focus groups were conducted between February and August 2021. Official customary approval was obtained from the traditional leaders in each community before the focus groups were held; a key customary requirement for community entry in most Ghanaian communities.^13^ Before each focus group, the research objectives and participants' information sheet were read out to participants in the English Language. A Twi translation was also read to members who could not communicate fluently in English. After responding to any questions, written consent was obtained from each participant before the first author commenced the study.

The focus group interview guide was based on Rifkin's spider‐gram methodological tool to explore the extent to which the communities were involved in implementing their respective district's community‐based health intervention.^20^, ^28^ The methodological tool considers five key indicators in relation to PPE: Needs assessment, Leadership, Organisation, Resource Mobilisation and Management. The first indicator, ‘needs assessment’, has been described as the process of identifying the health problem and relating this to the health needs of the community.^28^, ^29^ *Leadership* examines the authority of the programme and how it acts in the community's interests, particularly in terms of representation of poor and marginalised groups. The third indicator assesses engagement from the point of ‘organisation’. That is, how successfully the health programme integrates with other pre‐existing community structures. *Resource mobilisation* focuses on a community's commitment to the health programme through their contribution of resources (in various forms) and involvement in the allocation of the resources to support the health programme.^28^ Finally, the *management* indicator measures the management and decision‐making structures of the health programme.

The focus groups explored nine broad areas, including participants' views and understandings of the health intervention programme, the extent of community involvement in the selection, design and implementation of the health intervention, the community's involvement in decision‐making and feedback processes and the extent of integrating existing community structures into the health programme. Finally, participants were asked to rank their respective community's involvement in the health programme on a spider‐gram scale of 1–5 (with 1 being the lowest and 5 being the highest involvement).

### 2.4 Data analysis

Interviews conducted in Twi were directly transcribed from Twi to English by the first author. A native Twi speaker was occasionally contacted to assist with translating some of the technical Twi words. To preserve the anonymity of respondents,^30^ transcripts were deidentified, and all participants were assigned unique identifiers for the analysis. Thematic analysis was employed. This focused on the five dimensions of the spider‐gram methodological tool.^31^, ^32^ Transcripts were coded in NVivo Version 12 Plus.^33^ The systematic process of collecting and analysing these research data was duly documented.^30^, ^34^ This involved the second and third authors verifying the quality of data coding and reviewing interview transcripts to ensure data consistency and questioning the analysis process to assess for bias in relation to any preconceived ideas that may be influencing the analysis. A detailed research diary was used to record all the first author reflections within 12 h of each focus group.^35^ The results of the focus group discussions are presented in the next section.

### 2.5 Ethical approval

The study was approved by the University of Otago Ethics Review Committee (20/002). In addition, approval was obtained from the Ashanti Regional Health Directorate of the Ghana Health Service. Data collected were not linked to individual participants.

## 3 RESULTS

This section presents the focus group findings under each of the five spider‐gram dimensions. Overall, across the three districts, 26 people participated in the focus groups, made up of traditional leaders (*n* = 3); residents/opinion leaders (*n* = 3); community health nurses/public health officers/midwives (*n* = 4); CHMC–CHVs (*n* = 9); assemblymen/assemblywomen (*n* = 1); CHVs only (*n* = 3) and programme/community health coordinators (*n* = 3) (Table 1). The focus groups lasted an average of 1.5 h. In terms of gender, 77% of the participants were male and 23% female. In addition, 42% of participants had received tertiary education, 12% secondary and 46% junior high school. The average age of participants was 50 years (ranging from 30 to 70 years). The average length of time participants had served in their health role ranged from 6 to 29 years. All participants were fluent in the local language (Twi), which was the preferred language for the focus groups.

### 3.1 Needs assessment

Under needs assessment, the participants' views varied across the three programmes. For instance, in the CHPS programme, the participants revealed that the community played a major role in identifying the health need. However, their involvement in the programme design and implementation was very limited.

After our request for a clinic was approved by the District Assembly, they should have contacted us for our input. We did not receive any feedback from them until the contractor started the project. During the construction, we made suggestions, but they did not bother to alter their original design to suit our demands. (CHV and CHMC FG 1)

This was, however, contrary to the CMCH programme, where although the community was not involved in the identification of the health need, they were adequately consulted, and their suggestion was largely considered in the design and implementation of the health programme.

This was actually not one of the things we requested from the District Health Office. They brought it to us because, in their wisdom, our community was not doing well with the number of women who were dying or having complications giving birth. It was no news to us because many people in this community had experienced that. So, we all sat together and worked on how this programme would be successful; and they listened and worked with our advice, too. (CHV & CHMC member 1, FG 2)

Like the CMCH programme, participants from the Buruli Ulcer programme also expressed the view that the selection of the health programme was solely done by the health professionals rather than the community.

However, across the three programmes, it was noted that despite the limited involvement of the communities in identifying their health need, once the programme resonated with their health needs, it played a crucial role in gaining the community's support:

We needed the clinic badly. So irrespective of the fact that we were not listened to, we accepted and supported them throughout the construction to ensure this was completed. We needed something bigger than what they did for us, but half a loaf is better than none. (Traditional leader 1, FG 1)

Overall, from the three programmes, the participants ranked the community's involvement in ‘needs assessment’ at points 3, 2, 1 for the CHPS, CMCH and Buruli Ulcer programmes, respectively—generally indicating low PPE.

### 3.2 Leadership

In relation to leadership, participants across the programmes expressed varied views, particularly in terms of the representativeness of the CHMC on the programme. From the CHPS programme, participants explained that the formation of the CHMC was initially influenced by local politicians who appointed their political party followers to the committee with the expectation of receiving some financial benefits. As a result, the committee could not be described as entirely representative, although the CHMC played crucial roles in the implementation of the programme:

The party people brought their representatives to the committee. We don't know where they were selected from. They brought the names from the District Assembly Office and the District Health Office. Those of us who were instrumental in the establishment of the CHPS facility were initially taken out of the committee. I can say, only two out of the thirteen people were genuine selections. (CHV & CHMC member 1, FG 1)

Despite the CHMC/CHVs not being formally involved in the implementation of the CHPS programme, the committee still ensured an active interest in the programme to ensure the community's interest was considered in the implementation.

Different views were, however, expressed by the participants from the CMCH programme regarding the leadership of the programme. Both the community‐based participants and the health workers shared the views that the composition of the programme leadership was primarily dominated by the community representatives (CHMCs/CHVs), who also led the implementation of the entire CMCH programme. This was noted by all participants as the key factor that led to the successful implementation of the CMCH programme:

The implementation of the CMCH programme has really taught us a great lesson about how we can get the best out of communities. Just allowing them to lead the process and providing professional guidance to them definitely is the key. The CHMCs and the volunteers led the entire programme. (District CHPS coordinator FG 2)

While the CMCH programme was reported to have PPE in terms of leadership, it was apparent that in the Buruli Ulcer programme, CHVs did not have any decision‐making power as their role functioned within the mandated design of the programme—where the health professionals made key decisions.

The programme has a decision‐making structure that everyone from the national to the community follows. The CHVs are important but the community's decisions had to still go through the decision‐making structure of the programme. So yes, I can say they did not have so much autonomy in terms of making decisions. (BU coordinator, FG 3)

Lessons from the CMCH and the CHPS programmes showed that allowing communities or their representative to take active initiatives was key to achieving the aims of community‐based health programmes. For instance, it was noted from the focus groups that the community representative from the CMCH programme created a community zonal health system, where the community was divided into zones and a CHMC member and a CHV headed each zone. The community representatives regularly visited patients within their zones and provided reports on their visits in terms of the number of pregnant women, antenatal visits and child vaccinations, among other indicators. These reports provided to the health workers regularly helped monitor those patients' progress and identified key health issues at early stages. Thus, strong leadership, led by the community is regarded as key to achieving the major aims of such programmes:

The whole programme was a community‐led one. We the health workers did not come in so much. The District Health Directorate and the staff of the clinic empowered the community to be at the forefront of the programme to implement this. They took so many initiatives that ensured we end preventable maternal and child mortality in this community. We ourselves have been amazed at the results. When community people are empowered, they can really do so much. (Midwife, FG 2)

Overall, on the leadership dimension, the Buruli Ulcer programme was ranked the lowest 2, while the CHPS programme was ranked 4 and the CMCH was ranked 5.

### 3.3 Resource mobilisation

Our findings revealed that gaining community support for resource mobilisation is an indication of a community's support for the health intervention. An analysis of the data revealed that all three communities significantly contributed in ‘cash’ and in ‘kind’ to support the programmes. For instance, from the CHPS programme, it was found that despite the community being among the poorest in the district, an individual community member donated a piece of land for the clinic. In addition, the community mobilised various cash contributions to support the purchase of water reservoir tanks, water pumps and other necessary equipment needed to connect water to the health facility, which were not provided by the government as part of the construction of the clinic—which could have delayed the use of the clinic. In terms of ‘kind’ contributions, the findings revealed that while men in the community volunteered as construction site workers for the clinic, the women also fetched water from distant locations for the construction and provided free food for the clinic construction workers.

As for support, we did our best. This community came together to ensure this was a successful programme. One of our sub‐chiefs donated his personal piece of land for this CHPS facility. Apart from that, we also appealed for financial support from the community during our durbars to support the water project. We bought pumps, PVC pipes, poly tank [water reservoir], and many other things for the clinic. These were things government did not provide but the community did it ourselves through the funds we were able to generate both from the durbar and some prominent individuals from our community. (CHV & CHMC chairman, FG 1)

Similarly, from the CMCH programme, the study findings revealed that although the programme did not require considerable resources, the community still contributed substantially to the success of this programme—both in ‘cash’ and ‘kind’. These included cash donations mobilised from community durbars and from other individual members of the community to support the work of the CHVs and the CHMC towards the programme. Some parts of the funds mobilised were used to support pregnant women who could not afford the cost of their drugs and those who were not enroled in the National Health Insurance Scheme to be able to benefit from the national free maternal healthcare programme:

The amounts we mobilised were not much but at least it was enough to help enrol many pregnant women on the National Health Insurance Scheme, purchase some essential drugs for sick newborn babies, and also support the volunteers with mobile phone credits. (Assemblyman, FG 2)

While the user communities contributed significantly towards supporting the CHPS and the CMCH programmes, the Buruli Ulcer programme did not receive similar support from the community. Participants indicated that the programme managers ensured the provision of all the needed resources required for the programme and controlled its allocation. Although this contributed to the programme's immediate success, it failed to promote community ownership, which is critical for the long‐term sustainability of the programme. Again, while the CHMCs/CHVs in the CMCH programme had greater control of the allocation of the resources mobilised for the programme, the Buruli ulcer and CHPS programmes did not have any control over the allocation of those resources.

Ranking the resource mobilisation dimension on the spider‐gram, participants from the CHPS programme ranked their involvement at a scale of 4, CMCH—5 and Buruli ulcer—1.

### 3.4 Organisation

Our study findings showed that ‘organisation’ was the only spider‐gram dimension that received the highest PPE ranking across all three selected community‐based health interventions. The new programmes were successfully integrated into pre‐existing community structures. For instance, participants from the CHPS programme indicated that before the programme, the community had its traditional way of providing healthcare, particularly through the traditional birth attendants (TBAs) and traditional herbal healers. To avoid creating parallel structures that compete with the existing traditional community healthcare structures, the CHVs/CHMCs led the successful integration of the TBAs and the traditional herbal healers into the new programme. This included involving the only TBA in the community in the new programme by involving her in promoting antennal care as well as other nonclinical activities in the clinic. The traditional herbal healers were also supported with regular training to identify and quickly refer cases to the clinic for early detection and treatment. It was noted in the focus groups that not handling the integration process well could have resulted in conflict between the health workers and the traditional healthcare structures in the communities. Again, as most community members trust the traditional healthcare providers due to years of a close relationship with them as well as other cultural factors, there was still a possibility that the new programme could have been sabotaged if those pre‐existing community health structures were not integrated well. A Midwife explained further:

We knew of several stories like this in many communities, which usually leads to hospital workers refusing posting to certain communities in Ghana. It can be very dicey. So, when we came here and realised this was the challenge, we needed to approach it tactfully. Fortunately, having the husband of the TBA as a committee member was also good, as he persuaded the wife to dialogue with us on this. As I speak, she is here with us in the facility, assisting us in so many ways. As I earlier told you, if we had not used the best approach, the pregnant women would not have patronised the clinic because they trust and know TBA better than us. We did not condemn her practice but accepted her fully. We later exposed her to some of the possible reasons women die in her hands during childbirth. She accepted to work with us, and it has been cordial till date. (Midwife, FG 1)

Lessons from the CMCH and the Buruli Ulcer programmes also indicated that the programmes were implemented with consideration of existing CHMCs and CHVs in the communities. All TBAs within the community were trained and supported to refer cases to health professionals. In the case of the CMCH programme, each CHV and a Community Health Nurse were assigned the responsibility of supervising the overall work of the TBAs to ensure they complied with the agreed protocols. Participants also noted that the programmes integrated the work of traditional herbal healers, particularly those who provided herbal concoctions to newborns or Buruli ulcer patients. Although participants acknowledged this was not entirely successful as some did not cooperate, the awareness helped reduce neonatal mortalities and late detection of Buruli ulcer cases.

A participant from the District Directorate of Health Services elaborated further:

We have had good support from the locals, especially the TBAs. Most of them were aware they contributed to this problem so making them feel part of the efforts to resolve this was helpful. They did not feel threatened to lose their jobs. We rather empowered and trained them. We have only two midwives here so we cannot handle all the cases. They play important roles, so we needed to just empower them and let them refer early when they see the early warning signs. (District CHPS Coordinator, FG 2)

From the findings, it was apparent that implementing a community‐based health intervention programme through an existing community‐based health structure (including CHMC and CHVs) ensures the programme is responsive to the community's culture and values; moreover, the programme becomes acceptable to the larger community or users.

Across all three programmes, participants ranked ‘organisation’ dimension 5—meaning PPE was wide and excellent.

### 3.5 Management

On the last spider‐gram dimension of ‘management’, the findings varied across the three selected community‐based health interventions. In the Buruli Ulcer programme, it was revealed that although the CHVs were chosen democratically, the health programme, however, was not independently managed by the local community people or the CHVs. The management of the programme was entirely in the hands of the Buruli Ulcer programme managers, who took all key decisions. This was similar to the CHPS programme, where participants also indicated that the overall management of the CHPS programme was mainly in the hands of the District Health Directorate. As a result, decisions from CHMCs were subject to acceptance by health professionals. This, to a considerable extent, reduced the management influence of the CHMC on the CHPS programme. A participant highlighted this in the excerpt below:

The role we played as a community and CHVs was to support them because the programme was for our own good. Everything was in their hands. We only followed their direction and how they wanted us to support this programme. (CHV 1, FG 3)

While this allowed the health professionals to make decisions based on their expert knowledge, the community‐based participants expressed the view that most of these decisions did not consider their local needs and interests.

Contrary to the above findings, the CMCH programme, once again, provided a more significant lesson for the management of community‐based health intervention programmes by providing a broader PPE on this spider‐gram dimension. The participants indicated that the community's health committee largely managed the programme with little interference from the District Directorate of Health. Although the health need and programme design emanated from the District Health Directorate, its management was largely in the hands of the CHMC and CHVs:

You can see from our discussions that the community themselves managed the programme. We only provided guidance for their work. We stayed back and rather supported them through the CHMC and the CHVs and I think they did the entire work. We from the district health administration did not influence the management of the programme. (District CHPS coordinator, FG 2)

This seems to have played a key role in the successful implementation of the programme as well as sustaining the programme beyond its implementation phase. Particularly, the community members and their representatives were involved in most aspects of the programme implementation, as well as building their capacities to manage the programme beyond the implementation phase. This provided an easier option for sustaining such community‐based health programmes and promoting community ownership to ensure the gains achieved in improving maternal and child health outcomes in the community were sustained.

The Buruli ulcer and CHPS programmes, however, presented a different finding as the research found no clear plan in relation to building local capacities to sustain the programme beyond the implementation phase. For instance, findings from the Buruli Ulcer programme showed that there were limited efforts to train or build the capacities of the CHVs and other key community stakeholders to continue the Buruli ulcer control activities after the official programme funding had ended. It was noted from the focus groups that the programme design did not have a clear pathway towards sustaining the gains made as the programme relied on the generosity of a hospital to partly cater for the cost of patient treatment—which was however not comprehensive.

…. the sad reality is that the programme has now run out of funds, and we rely on the benevolence of the [………] Hospital here to provide free treatment of healthcare to our confirmed Buruli Ulcer patients. Now, it is difficult supporting the activities of the CHVs and even other community surveillance and health promotion activities. (BU Coordinator, FG 3)

**Figure 1:**
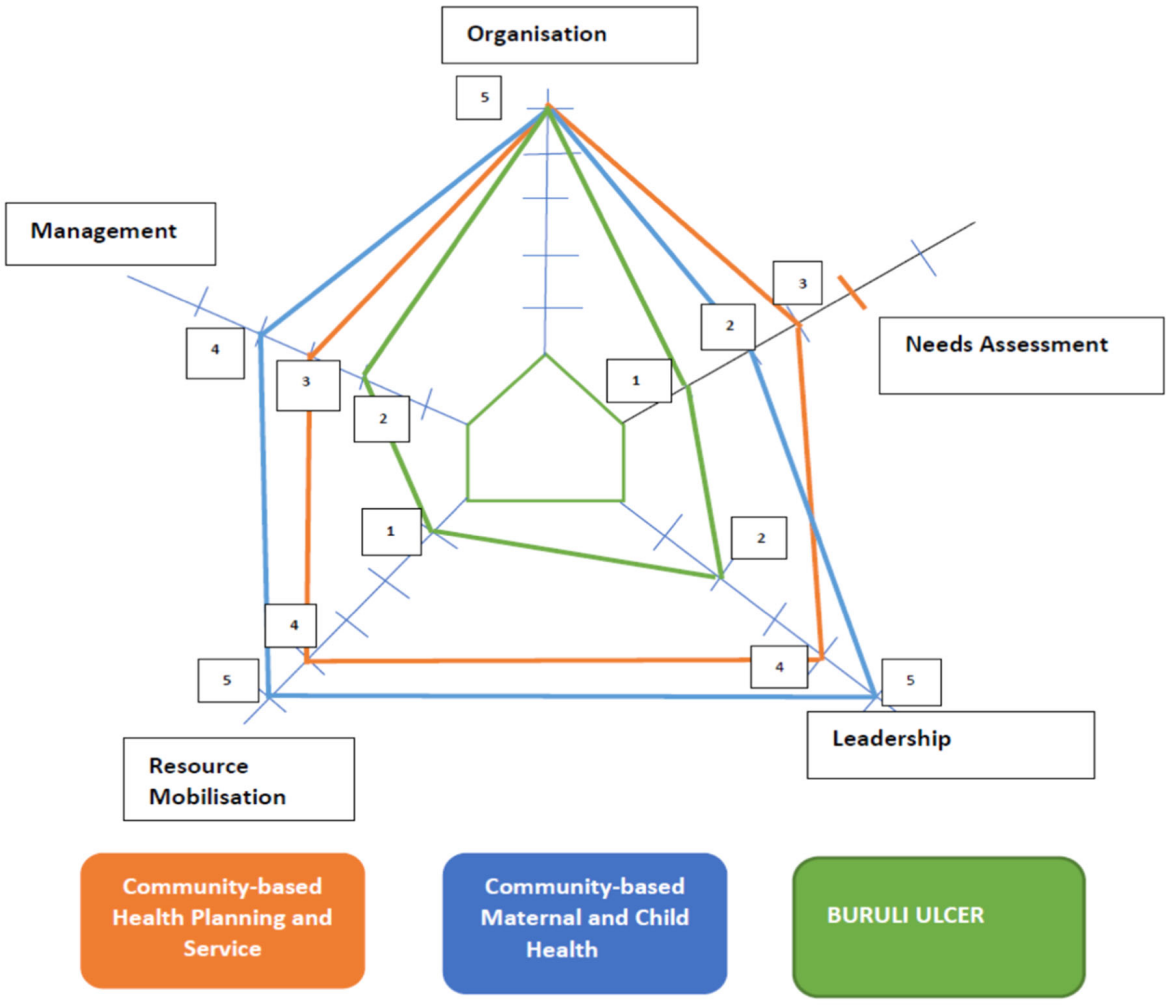
Spider‐gram assessing the level of PPE in the selected community‐based health programmes. PPE, patient–public engagement.

Overall, the participants ranked the community's level of participation in relation to ‘management’ at Point 3 for the CHPS programme, Point 4 for CMCH and Point 2 for the Buruli Ulcer programme. A summary of the rankings for the three interventions is depicted in Figure 1.

## 4 DISCUSSION

This research provides an in‐depth understanding of the role of PPE in improving the design and implementation of community‐based health interventions. Three community‐based health intervention programmes were selected: CHPS, CMCH and Buruli ulcer. The spider‐gram analytical tool was used in each focus group to assess the extent of PPE in the design and implementation of the three community‐based health intervention programmes.

The findings from this study highlighted a range of factors that could enhance the success of community‐based health interventions through the specific implementation of PPE. Across the three community‐based health programmes (Figure 1), there was limited community involvement in the needs assessment phase in advance of introducing a programme. That is, most communities were not involved in selecting the health needs. Notably, from the CHPS and Buruli Ulcer programmes, our research found that the community's involvement in the programme design and implementation was also limited, and this negatively affected the programme goals and sustainability. Members of the CHMC/CHV largely felt most of their concerns were ignored in the design and implementation of the programme. For instance, in the CHPS programme, the participants cited an instance where the programme implementers ignored the suggestion of the CHMC to construct separate clinical wards for both male and female patients. The failure to heed this suggestion seems to have affected patronage of the clinic—as the community largely considered this culturally unacceptable for people of the opposite gender to be sharing the same room.^36^ While this drastically affected attendance at the clinic, it also negatively impacted the overall aim of the programme. Thus, as argued by Pronk et al.,^19^ this act of unilaterally selecting, designing and implementing health programmes with limited community involvement is inimical to achieving optimal PPE as well as achieving the best outcomes in community‐based health interventions.^37^ It also denies the community representatives the power to influence decision‐making about the programmes and, therefore, becomes less empowered to engage in decisions that promote the people's interest.^38^, ^39^ Notwithstanding the limited community involvement in needs assessment, our study noted that the selected health programmes did resonate with the communities' health needs, which is also key in gaining community support for the programme.

Across all programmes, the community representatives were independently selected by the communities although it was noted that the initial formation of the CHMCs/CHVs was sometimes influenced by high‐status people, particularly politicians who imposed their political party members on the committee. Specifically, from the CHPS programme, it was observed that the imposed members had contributed to the failure of the programme to meet the needs of the community, as their imposition was at a crucial time when the community needed a strong voice to make changes to the programme design. Using Arnstein's terminology, they were ‘rubberstamp advisory committee members’ whose involvement in the programme was to satisfy the CHPS policy requirement of having a CHMC in place but seemed to prioritise the bidding of their political masters rather than influencing decision‐making for the benefit of the community.^40^, ^41^ Contrary to the CHPS and Buruli Ulcer programmes, where the CHMCs/CHVs did not have much control over certain aspects of the programmes, the community representatives from the CMCH programme were found to be very active and took various initiatives to help achieve the key aims of the programme. For instance, the CHMCs/CHVs in the CMCH programme were innovative in creating a community zonal health system where the community was divided into zones and a CHMC member and a CHV headed each zone. This ensured that every pregnant woman within the zones was regularly visited and reported on by their zonal CHMC/CHV to the health workers for early medical interventions. This was found to have significantly reduced maternal mortalities and other obstetric complications. This finding supports Charles and DeMaio's^2^ model, which argues that providing sufficient decision‐making power to lay representatives (CHMCs and CHVs) enables them to exhibit strong leadership, which is key to achieving the overall aim of health initiatives.

Furthermore, on the leadership dimension, one key aspect we found to have affected the implementation of community‐based health programmes was the male‐dominant nature of the community representatives as seen in the CHPS and the Buruli Ulcer programmes. In the specific case of the Buruli Ulcer programme, it was observed that not including female CHVs affected the programme because the male CHVs were largely restricted from physically examining suspected female Buruli ulcer patients as this was interpreted in the community's culture to mean inappropriate ‘touch’. However, this could have been avoided if female CHVs had physically examined such patients. This finding is also consistent with a similar study conducted in the Upper West Region of Ghana, where the male‐dominant CHVs were restricted in engaging female community members due to other cultural barriers.^20^

Regarding resource mobilisation, this research observed that across the three programmes, the communities significantly contributed various resources (cash, materials and labour) to support the programmes. While this reflected the utilitarian value of the health programmes to the communities, there were few efforts by the programme implementers to develop local capacities to ensure community ownership and support for post‐implementation sustenance. Yeboah and Jagri^42^ similarly found that in a CHPS study conducted in the Central Region of Ghana,  the programme could not be sustained after implementation due to limited community involvement. As rightly argued by Charles and DeMaio,^2^ limited community involvement in decision‐making affects the ability to build a strong local capacity for post‐implementation management. Nonetheless, our study notes that there is a greater possibility that when communities are highly involved in programme design and implementation, they are more likely to support the programme, especially if it resonates with their healthcare needs. This confirms an earlier assertion that PPE could become an important means through which governments could explore providing adequate resources for community‐based health activities, especially in a resource‐constrained country like Ghana.^43^

Another important lesson drawn from the three community‐based health intervention programmes was the successful integration of pre‐existing community health structures into the new health programme. Anecdotal evidence in Ghana shows that these pre‐existing community health structures, like the TBAs, herbal and spiritual healers pose a significant threat to the successful implementation of community‐based health programmes.^44^, ^45^ Other studies have also reported clashes between health professionals and many of these pre‐existing community health structures—leading to some health workers refusing postings to such communities over threats to their lives.^46^, ^47^ However, lessons from across the three programmes showed that implementing PPE by allowing CHMCs/CHVs to lead the process ensures the successful integration of TBAs, herbal practitioners and spiritual healers into the new programme. As part of the integration process, the PPE process provided adequate training and logistical support to the TBAs, herbal practitioners and spiritual healers, enabling them to manage or refer medical cases early to the clinic effectively. Again, the implementation of PPE ensured that the community‐based health intervention programmes did not compete with these pre‐existing community health structures but instead worked together to ensure the success of the new health programme. In a similar study conducted in New Zealand, it was found that integrating the pre‐existing community structures into the new community health programme was key to avoiding parallel running of health structures that compete with each other, thereby affecting the success of community‐based health interventions.^48^

Finally, in examining the role of PPE in relation to management, our study found that community representatives, despite their more significant contributions to the success of community‐based health programmes, have mostly not been involved in the management of such programmes. Scholars like Bate and Robert^49^ have suggested codesigned and comanaged health programmes between communities and health authorities to improve outcomes. However, our findings revealed that the implementation of the CHPS and Buruli Ulcer programmes, particularly, were found to have provided limited community involvement in managing these programmes. This greatly affected the sustainability of the two programmes, especially after their funding ended. Contrary to the CHPS and Buruli Ulcer programmes, the CMCH programme provided a wide and excellent community involvement in the programme's management. This is greatly reflected in the outcome of the programme as both the district and community‐level participants reported that the CMCH programme achieved one of the best and most sustained outcomes in terms of maternal and child health indicators compared to the previously implemented Millennium Accelerated Framework programme, which in spite of having more human and financial resources achieved only a temporary success. The present and earlier findings confirm the need to widely involve and empower communities in the design and implementation of health programmes to ensure continued sustainability beyond the implementation phase.^41^, ^42^, ^50^

Despite this study providing a fundamental understanding of the role of PPE in improving outcomes in community‐based health interventions, it still has some key limitations. First, the Spider‐gram does not include an opportunity for an analysis of context. Thus, measuring PPE levels and analysing the study findings according to the spider‐gram framework appears very subjective. However, the rankings from the participants largely reflected our study findings. Second, the views presented in this paper reflect only the personal experiences of a few participants in three selected districts in Ghana. These participants mainly had roles linked to the selected community‐based health interventions. Therefore, it is possible they may had a vested interest in either promoting or discrediting the programme. In spite of these limitations, our study has provided an in‐depth understanding of the significant role of implementing PPE to improve outcomes in community‐based health interventions.

## 5 CONCLUSION

Overall, we note that implementing PPE in community‐based health interventions is necessary to improve the poor health outcomes in many Sub‐Saharan African countries, particularly Ghana. The findings of this study suggest that widely involving user communities or their representatives in the design and implementation of community‐based health intervention significantly improves the aims of these programmes. Therefore, we recommend the need to avoid tokenistic approaches to PPE by ensuring that user communities are genuinely involved in designing and implementing community‐based health interventions as well as being considered in decision‐making. Finally, to help sustain such health programmes beyond the implementation phase, we suggest the need to build the capacities of the local communities or their representatives by involving them in all stages of the programme design and implementation.

## AUTHOR CONTRIBUTIONS

**Conceptualisation**: Samuel E. Ankomah, Adam Fusheini and Sarah Derrett. **Methodology**: Samuel E. Ankomah, Adam Fusheini and Sarah Derrett. **Software**: Samuel E. Ankomah. **Validation**: Adam Fusheini, Sarah Derrett. **Formal Analysis**: Samuel E. Ankomah. **Investigation**: Samuel E. Ankomah. **Resources**: Sarah Derrett. **Data Curation**: Samuel E. Ankomah, Adam Fusheini and Sarah Derrett. **Writing—original draft**: Samuel E. Ankomah. **Writing, review and editing**: Adam Fusheini, Sarah Derrett. **Supervision**: Adam Fusheini and Sarah Derrett. **Project administration and funding acquisition**: Samuel E. Ankomah and Sarah Derrett.

## CONFLICT OF INTEREST STATEMENT

The authors declare no conflict of interest.

## Data Availability

The data that support the findings of this study are available from the corresponding author upon reasonable request.
